# Ablation of retinal ciliopathy protein RPGR results in altered photoreceptor ciliary composition

**DOI:** 10.1038/srep11137

**Published:** 2015-06-11

**Authors:** Kollu N. Rao, Linjing Li, Manisha Anand, Hemant Khanna

**Affiliations:** 1 Department of Ophthalmology, University of Massachusetts Medical School, Worcester, MA 01605, USA

## Abstract

Cilia regulate several developmental and homeostatic pathways that are critical to survival. Sensory cilia of photoreceptors regulate phototransduction cascade for visual processing. Mutations in the ciliary protein RPGR (retinitis pigmentosa GTPase regulator) are a prominent cause of severe blindness disorders due to degeneration of mature photoreceptors. However, precise function of RPGR is still unclear. Here we studied the involvement of RPGR in ciliary trafficking by analyzing the composition of photoreceptor sensory cilia (PSC) in *Rpgr*^*ko*^ retina. Using tandem mass spectrometry analysis followed by immunoblotting, we detected few alterations in levels of proteins involved in proteasomal function and vesicular trafficking in *Rpgr*^*ko*^ PSC, prior to onset of degeneration. We also found alterations in the levels of high molecular weight soluble proteins in *Rpgr*^*ko*^ PSC. Our data indicate RPGR regulates entry or retention of soluble proteins in photoreceptor cilia but spares the trafficking of key structural and phototransduction-associated proteins. Given a frequent occurrence of *RPGR* mutations in severe photoreceptor degeneration due to ciliary disorders, our results provide insights into pathways resulting in altered mature cilia function in ciliopathies.

Cilia are microtubule-based antenna-like extensions of the plasma membrane in nearly all cell types, which regulate diverse developmental and homeostatic functions, including specification of left-right asymmetry, cardiac development, renal function and neurosensation^1^,^2^. Cilia formation is initiated when the mother centriole (also called basal body) docks at the apical plasma membrane and nucleates the assembly and extension of microtubules in the form of axoneme. Distal to the basal body, cilia possess a gate-like structure called the transition zone (TZ), which is thought to act as a barrier for allowing selective protein cargo to enter the axoneme microtubules by a conserved process called intraflagellar transport^3^,^4^,^5^. Defects in cilia formation or function result in severe ciliopathies, ranging from developmental disorders, including mental retardation, disruption of left-right asymmetry and skeletal defects to degenerative diseases, such as renal cystic diseases and retinal degeneration due to photoreceptor dysfunction^6^,^7^.

Photoreceptors develop unique sensory cilia in the form of light-sensing outer segment (OS). The OS, in addition to the ciliary membrane, consists of membranous discs loaded with photopigment rhodopsin and other proteins such as peripherin/rds and rod outer membrane protein ROM1^8^,^9^. The region between the basal body and the distal cilium is called TZ or connecting cilium of photoreceptors 8,9,10. Defects in TZ structure and function result in altered trafficking of proteins to the OS, leading to photoreceptor degenerative diseases, such as Retinitis Pigmentosa (RP)^11^.

RP is a genetically and clinically heterogeneous progressive hereditary disorder of the retina^12^. X-linked forms of RP (XLRP) are among the most severe forms and account for 10–20% of inherited retinal dystrophies. XLRP is characterized by photoreceptor degeneration, with night blindness during the first or second decade, generally followed by significant vision loss by fourth decade^13^. Mutations in the ciliary protein retinitis pigmentosa GTPase regulator (*RPGR*) account for >70% of XLRP cases and 15–20% of simplex RP cases^14^,^15^,^16^,^17^,^18^,^19^,^20^. *RPGR* mutations are also reported in patients with atrophic macular degeneration, sensorineural hearing loss, respiratory tract infections, and primary cilia dyskinesia^19^,^20^,^21^,^22^,^23^,^24^.

RPGR localizes predominantly to the TZ of photoreceptor and other cilia^25^,^26^ and interacts with TZ-associated ciliary disease proteins^26^,^27^,^28^,^29^,^30^,^31^. Studies using animal models indicate that *Rpgr* ablation or mutation results in delayed yet severe retinal degeneration^32^,^33^,^34^,^35^. However, the precise function of RPGR and the mechanism of associated photoreceptor degeneration are poorly understood. In this report, we sought to assess the role of RPGR in ciliary trafficking by testing the effect of loss of RPGR on the composition of the photoreceptor sensory cilia in mice. Our results suggest that RPGR participates in maintaining the function of mature cilia by selectively regulating (directly or indirectly) trafficking of proteins involved in distinct yet overlapping pathways.

## Results

### Purification of photoreceptor sensory cilium (PSC)

**Figure 1:**
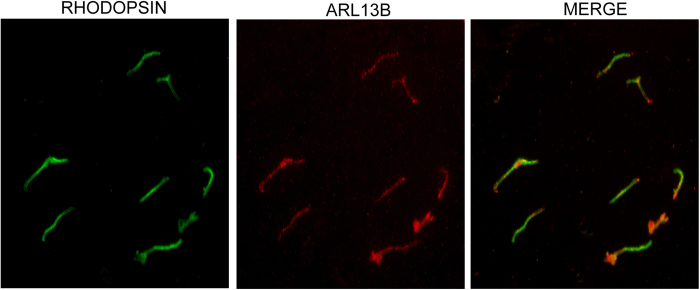
Immunofluorescence analysis of isolated PSC: PSC was isolated from mouse retina and stained with anti-rhodopsin (green) and anti-ARL13B (red) antibodies. Merge shown co-localization of rhodopsin with the ciliary marker.

**Figure 2:**
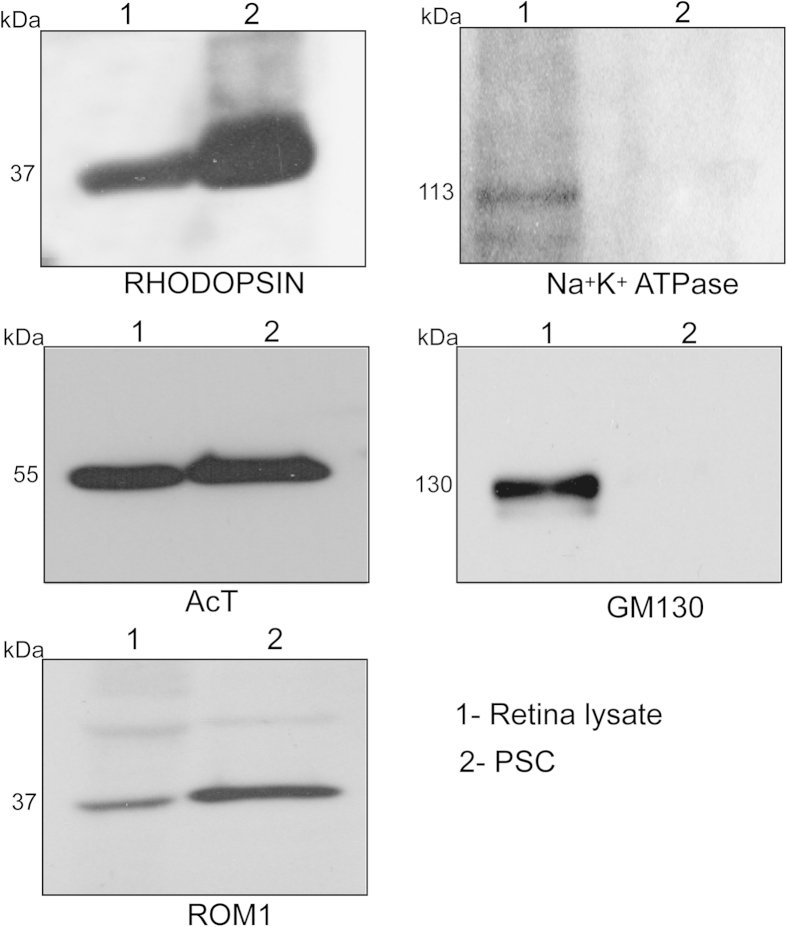
Purity of the PSC: PSC fraction was analyzed by SDS-PAGE and immunoblotting using indicated antibodies. Lane 1: total retina lysate; Lane 2: PSC. Molecular weight markers are shown in kilo Daltons (kDa). Enrichment of known ciliary proteins was detected whereas cytosolic proteins were undetectable in the PSC.

We and others previously showed that the *Rpgr*^*ko*^ mice exhibit photoreceptor degeneration starting at around 6 months of age^32^,^35^. Based on this information, we selected two stages of *Rpgr*^*ko*^ mice to assess PSC composition: 2 months and 4 months. We hypothesized that (i) these stages would represent changes in protein trafficking prior to onset of degeneration and (ii) progression in the changes observed from 2 to 4 months of age are likely candidates for true disease-associated defects. We used age-matched wild-type littermates as controls. The retinas were isolated and subjected to sub cellular fractionation as described in the Methods section. Fluorescence microscopic analysis of our PSC preparations using anti-rhodopsin and ciliary marker ARL13B (ADP-ribosylation Factor like 13B) showed that the fractions were relatively pure sensory cilia (Fig. 1). To further validate the purity of the PSC fractions, we carried out immunoblot analysis using antibodies against marker proteins residing in cytosol (inner segment), mitochondria, as well as in the cilia. As shown in Fig. 2, PSC fraction was enriched in sensory cilia proteins rhodopsin, acetylated α-tubulin and rod outer membrane (ROM1) protein but not in inner segment localized proteins GM130 (Golgi marker) and Na^+^K^+^ATPase (a mitochondrial marker).

### Proteomic analysis of PSC

To test the role of RPGR in regulating protein trafficking into the cilia, we assessed the composition of the PSC of the *Rpgr*^*ko*^ mice as compared to wild type mice. We performed MS/MS analysis of the PSC at 2 months and 4 months of age (Supplementary Tables S1 and S2 online, respectively). Each experiment consisted of 6 retinas from wild type and 6 retinas from *Rpgr*^*ko*^ mice. Data are average of three biological replicates at each age. At 2 months of age, we detected 1366 proteins in wild type and *Rpgr*^*ko*^ PSC. At 4 months, we detected a total of 1614 proteins in wild type and *Rpgr*^*ko*^ PSC. As predicted, RPGR was not detected in the *Rpgr*^*ko*^ PSC.

We then analyzed our MS/MS data to identify proteins that were increased or decreased in the in the *Rpgr*^*ko*^ PSC as compared to wild type and categorize them based on their relative abundance. To this end, we applied the following criteria: (i) the average emPAI values had to be <0.5 fold if the abundance of the protein decreased and >2 fold if abundance increased and (ii) the number of unique peptides had to be greater than 3, if a protein is not detected in the other genotype. Based on these criteria, the proteins that increased or decreased are listed in Tables 1, 2, 3, 4. At 2 months of age, we detected 28 proteins that decreased in amount by less than 0.5 fold in *Rpgr*^*ko*^ PSC as compared to wild type. On the other hand, we detected 30 proteins at 4 months of age that showed less than 0.5-fold reduction in *Rpgr*^*ko*^ PSC. Among the category of proteins whose relative abundance increased by >2-fold in *Rpgr*^*ko*^ PSC at 2 months of age, we detected 17 proteins, whereas at 4 months of age, we found 24 proteins in *Rpgr*^*ko*^ PSC.

It is becoming clear that the TZ also acts as a size-exclusion barrier for soluble proteins^36^,^37^,^38^. Interestingly, soluble proteins that reduced in abundance by 0.5-fold in *Rpgr*^*ko*^ PSC decreased from 13 out of 28 at 2 months of age (46%; average molecular weight: 51.7 kDa) to 6 out of 30 at 4 months of age (20%; average molecular weight of 80 kDa) (Table 5). Whereas 2 out of 17 proteins that increased in amount at 2 months of age (11.7%; average molecular weight of 99.5 kDa) are soluble proteins, 7 out of 24 proteins increased in amount at 4 months of age (~30%; average molecular weight of 99 kDa) are soluble proteins (Table 5). We therefore, suggest that the barrier for entry or retention of soluble proteins into the PSC is affected in the absence of RPGR.

### Phototransduction proteins and known ciliopathy-associated proteins in *Rpgr* ^*ko*^ PSC

Previous studies showed a reduction in photoreceptor function in the *Rpgr*^*ko*^ mice^32^. We therefore, tested if this phenotype was due to an earlier defect in reduced translocation of selected phototransduction proteins to the OS. Our MS/MS analysis as well as validation by immunoblotting revealed no significant difference in the amount of arrestin, ROM1, and cyclic nucleotide gated channel CNGB1 in the *Rpgr*^*ko*^ PSC as compared to wild type mice. These results suggest that photoreceptor dysfunction in the absence of RPGR in mice is not likely due to a defect in the trafficking of the tested phototransduction-associated proteins.

Alterations in BBS-associated proteins are associated with photoreceptor dysfunction among other ciliopathy disorders. Our analysis revealed BBS1, BBS2, BBS5, and BBS7 in the PSC; however, their levels did not alter between age-matched wild type and *Rpgr*^*ko*^ PSC. Similarly, we did not detect differences in IFT polypeptides or associated Kinesin motor subunits in the *Rpgr*^*ko*^ PSC.

### Proteins with altered abundance in *Rpgr* ^*ko*^ PSC

**Figure 3:**
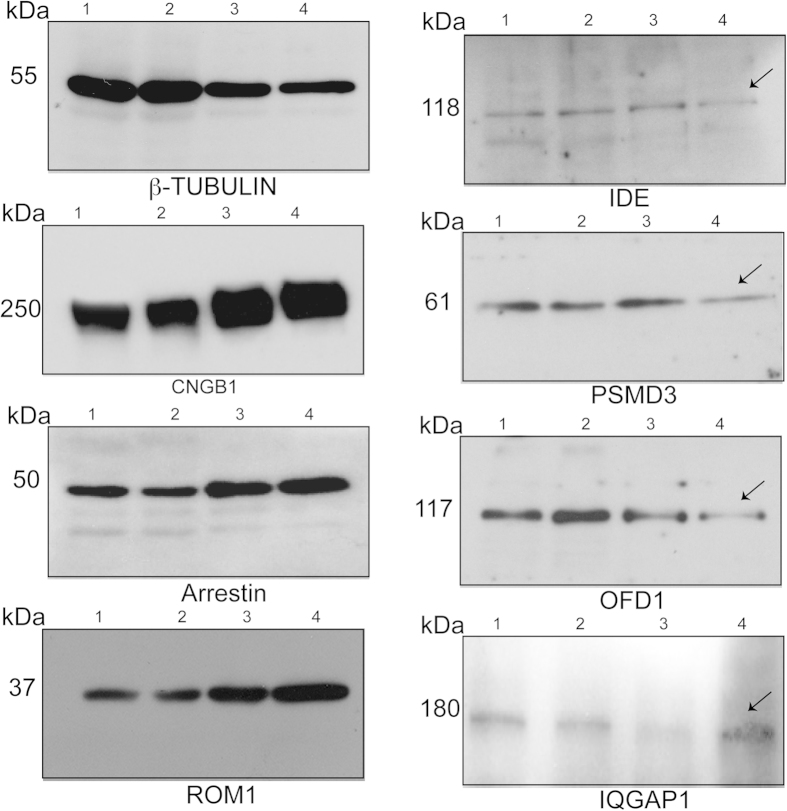
Validation of selected proteins identified by MS/MS analysis. PSC from wild type (WT) and *Rpgr*^*ko*^ mouse retina were analyzed by SDS-PAGE and immunoblotting using indicated antibodies. Lanes 1 and 2: total retina lysate from WT and *Rpgr*^*ko*^ mice, respectively; Lanes 3 and 4: PSC from WT and *Rpgr*^*ko*^ mice, respectively. Arrows indicate altered proteins in the *Rpgr*^*ko*^ PSC. Molecular weight markers are shown in kilo Daltons (kDa).

A majority of proteins that decreased in amount in *Rpgr*^*ko*^ PSC at both stages belonged to the category of ubiquitin-proteasome system (UPS) and cilia function (Tables 1 and 2 and Supplementary Figure S1). As the UPS is enriched at the cilium and has been detected in the PSC in earlier proteomics analyses (discussed later)^10^,^39^, we selected PSMD3 (26 S proteasome non-ATPase regulatory subunit 3) and Insulin degrading enzyme (IDE) for further analysis. Immunoblot analysis of PSC from wild type and *Rpgr*^*ko*^ showed a reduction in the amount of both PSMD3 and IDE1 in the *Rpgr*^*ko*^ PSC (Fig. 3). Total amount of these proteins did not alter, indicating that there was no change in the expression levels of PSMD3 and IDE in *Rpgr*^*ko*^ retina. It was recently shown that proteasomal function is modulated by ciliary-centrosomal protein OFD1^39^. As we detected reduced abundance of OFD1 in the *Rpgr*^*ko*^ PSC at 4 months of age, we performed immunoblot analysis using anti-OFD1 antibody. Our results validated the MS/MS data and showed reduced amount of OFD1 amount in *Rpgr*^*ko*^ PSC (Fig. 3). No change in the total protein levels of OFD1 was detected in these analyses.

We found high representation of the category of membrane trafficking and intracellular transport among the proteins that were increased in *Rpgr*^*ko*^ PSC (Tables 3 and 4 and Supplementary Figure S2). Specifically, we detected more than 5-fold increase in IQ-domain GTPase Activating Proteins IQGAP1, which is predicted to be involved in intracellular trafficking and neuronal regulation^40^. Given a crucial role of small GTPases and their regulation in the maintenance of PSC^28^,^41^,^42^, we validated the levels of IQGAP1 by immunoblot analysis. We found that indeed, amount of IQGAP1 is increased in the *Rpgr*^*ko*^ PSC relative to the wild type (Fig. 3). No change in the total levels of IQGAP1 was detected.

### Localization of altered proteins in the retina

**Figure 4:**
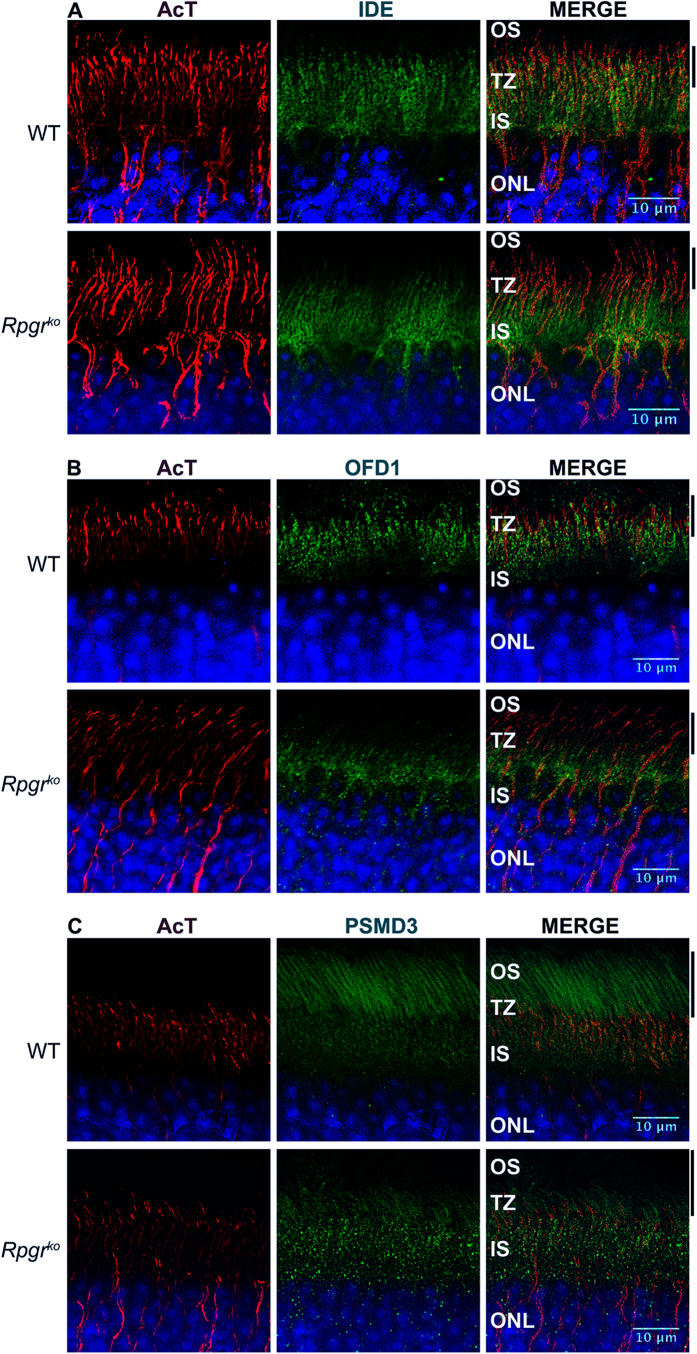
Localization of PSC proteins in mouse retina. Wild type (WT) and *Rpgr*^*ko*^ retinal sections were stained with indicated antibodies. Nuclei (blue) are stained with Hoechst. OS: outer segment; TZ: transition zone; IS: inner segment; ONL: outer nuclear layer; AcT: acetylated α-tubulin. Scale bar: 10 μm. Solid lines indicate the area that is stained by the respective antibody (green) in WT but not in *Rpgr*^*ko*^ retina.

Although all the proteins identified in our dataset have been previously reported as part of the mouse PSC proteome^10^, the localization of IDE, PSMD3, OFD1 and IQGAP1 has not been examined in mammalian retina. To corroborate our findings, we performed immunofluorescence analysis of these proteins using adult mouse retina. Our analysis revealed that in addition to the inner segment (IS) and outer plexiform layer (OPL), IDE, PSMD3, OFD1, and IQGAP1 localize to the TZ, as determined by co-staining with ciliary markers acetylated α-tubulin (AcT) or detyrosinated tubulin (DeTyr) (Fig. 4 and Supplementary Figure S3). Interestingly, PSMD3 was also detected in the OS. Consistent with a prominent role of OFD1 at the basal body, OFD1 co-localizes with γ-tubulin in photoreceptor IS (Supplementary Figure S3).

We next examined the effect of loss of RPGR on the localization of these proteins. Immunofluorescence analysis of age-matched *Rpgr*^*ko*^ mouse retinas revealed interesting observations (Fig. 4). We found reduced IDE and OFD1 associated signal reduced in the TZ with a concomitant mislocalization in the IS and outer nuclear layer (ONL) of *Rpgr*^*ko*^ retina (Fig. 4A,B). PSMD3 staining revealed strikingly reduced staining in the OS and increased in TZ/IS of *Rpgr*^*ko*^ retina (Fig. 4C).

## Discussion

Although RPGR is widely expressed and regulates ciliary trafficking, it is puzzling that RPGR mutations in humans do not result in typical cilia-associated systemic and developmental or early-onset defects. Rather, *RPGR* mutations are one of the most common causes of severe photoreceptor degenerative diseases of adulthood^19^,^43^. Photoreceptor cilia are unique with respect to their demands of maintenance of ciliary function. They undergo periodic shedding of distal tips of cilia with concomitant renewal of membrane and proteins at the base. It is estimated that about 10% of the OS tips are shed each day^44^,^45^. This is accompanied by massive transport of opsin and other OS proteins to the cilium^46^,^47^. Hence, it is conceivable that maintenance of ciliary function plays a crucial role in photoreceptor health. Our findings suggest that RPGR is one such regulator of functional maintenance of mature photoreceptors. RPGR accomplishes this task by likely modulating the docking and trafficking of key proteins involved in proteasomal function and intracellular protein trafficking.

Selecting 2 and 4 months of age of *Rpgr*^*ko*^ mice provided a unique opportunity to ascertain defects in photoreceptor protein trafficking that are likely to cause photoreceptor degeneration rather than a secondary effect of degeneration. Earlier studies using immunofluorescence analyses had revealed mistrafficking of opsins in *Rpgr*^*ko*^ retina at 6 months of age^32^. Our results indicate that loss of RPGR may not directly affect trafficking of opsin or other phototransduction proteins and the mislocalization of opsin observed in the *Rpgr*^*ko*^ retina could have been an effect of degenerating photoreceptors. Support of this hypothesis comes from several previous studies showing opsin mislocalization as a common phenotype across multiple gene mutations associated with retinal degeneration due to photoreceptor dysfunction^48^.

Membrane proteins targeted to cilia may dock at the periciliary membrane and then are loaded onto axonemal cargo carriers for delivery to the OS via the TZ^38^. However, fate of soluble proteins is not completely understood. It was reported that small soluble molecules such as GFP could equilibrate between inner and outer segments in frog photoreceptors^49^. Moreover, it was shown that arrestin and transducin diffuse through the TZ^50^,^51^,^52^. These findings suggest an absence of a diffusion barrier in photoreceptors, at least for smaller proteins. We detected a specific alteration in the levels of soluble proteins in the *Rpgr*^*ko*^ PSC. We found an overall increase in higher molecular weight soluble proteins at 4 months of age as compared to 2 months. As ciliary base is considered analogous to the nuclear pore complex and the role of another retinal ciliopathy protein RP2 in maintaining the function of nuclear pore proteins at the base of cilia^53^,^54^, it is conceivable that there is a size-exclusion barrier of soluble proteins at the TZ of photoreceptors. This barrier is likely maintained by RPGR and its interacting proteins. Additional analyses of the effect of RPGR and its multiprotein complexes on the integrity of such a barrier should provide crucial insights into the precise role of TZ of cilia.

Our data point to an important role of UPS in the pathogenesis of RPGR-associated photoreceptor degeneration. In addition to identifying several UPS components that show varying levels in *Rpgr*^*ko*^ PSC as compared to wild type, validation of three proteins that are directly or indirectly involved in UPS provide further support to the association of the UPS in cilia-dependent XLRP pathogenesis. We provide four scenarios to support this hypothesis: (i) among the UPS components identified in our PSC proteome, PSMD3 showed reduced levels at both 2 and 4 months of age in the *Rpgr*^*ko*^ PSC. A previous report on the involvement of another PSMD subunit PSMD13 in modulating RPE65 levels to regulate retinal health further supports the role of PSMD proteins in retinal health^55^. Moreover, UPS dysfunction has been reported in several retinal degenerative diseases^56^. The UPS is concentrated at the PSC and is implicated in regulating levels of phototransduction and other PSC proteins to cope with immense oxidative stress in photoreceptors^10^. Decrease in PSMD3 levels indicates that *Rpgr*^*ko*^ photoreceptors have reduced ability to cope with oxidative stress that accumulates over time; (ii) the UPS regulates insulin signal transduction by modulating degradation of key components, such as insulin receptor substrates^57^. Insulin, in turn, can inhibit proteasome and this action is dependent upon IDE^58^, which is also reduced in the *Rpgr*^*ko*^ PSC; (iii) PSMD3 modulates insulin resistance in association with polyunsaturated fatty acids (PUFAs)^59^, which are a key component of photoreceptor OS membranes^60^,^61^,^62^; (iv) OFD1, which is also reduced in *Rpgr*^*ko*^ PSC, is shown to modulate proteasome function^39^. Given that an intronic mutation in OFD1 is associated with XLRP^63^, we reckon that RPGR and OFD1 play overlapping roles in the manifestation of XLRP pathogenesis. Validation of these hypotheses warrants further investigations.

Vesicular trafficking by small GTPases in photoreceptors is critical for their function and survival^28^,^41^. Our analyses revealed abundant quantities of small GTPase regulators, such as IQGAP1 in the PSC of *Rpgr*^*ko*^ mice. IQGAPs are not only involved in intracellular trafficking, they are also implicated in actin-microtubule interaction and regulation of cytoskeletal dynamics. Commensurate with this, our analysis revealed alterations in levels of proteins belonging to the families of cytoskeleton-based vesicular trafficking to cilia. These results corroborate previous findings that RPGR regulates protein trafficking likely by modulating the activity of GTPases or regulators of GTPase activity^28^.

It should be noted that there is not much overlap between proteins whose abundance changes between 2 and 4 months. We posit that the proteins that show increased abundance at 2 months in the PSC may not be as abundant at 4 months due to progression of disease condition and associated subtle variations that may affect their abundance in the PSC in *Rpgr*^*ko*^ mice. Hence, such proteins would not make the cutoff of the threshold in our analysis. On the other hand, proteins that show reduced abundance at 2 months but are no longer detected at 4 months may reflect reduction in overall expression levels of such proteins. This would reduce to such an extent even in a wild type mouse retina that the variations are no longer significant in *Rpgr*^*ko*^ are and hence, do not make the cut off in our analyses.

Regulators of protein trafficking, such as RPGR are involved in maintaining the structure and function of mature cilia. Such functions are specifically crucial in mature neurons, which are under immense oxidative stress. Defects in RPGR result in subtle but incremental insult, which result in neurodegeneration. Our studies thus, provide clues to understanding pathways involved in the maintenance of mature cilia, which will also assist in delineating the pathogenesis of retinal degeneration in systemic ciliopathies.

## Methods

### Mice

*Rpgr*^*ko*^ mice (in C57BL6/J background) were procured from Dr. Tiansen Li (National Eye Institute) and were characterized earlier^32^. Control C57BL6/J mice were obtained from The Jackson Laboratories (Bar Harbor, ME). Both strains of mice were reared in the same animal facility at UMASS Medical School. All methods were carried out in accordance with the approved guidelines. All experimental protocols were approved by Institutional Animal Care and Use Committee and Institutional Biosafety Committee of UMASS Medical School.

### Antibodies

We procured antibodies against IDE and PSMD3 from Genetex (Irvine, CA); rhodopsin and IQGAP1, from EMD Millipore (Billerica, MA) and; anti-OFD1, anti-detyrosinated tubulin and anti-GM130 from Abcam (Cambridge, MA). Anti-acetylated α-tubulin and γ-tubulin were obtained from Sigma (St. Louis, MO); anti-ARL13B was obtained from Proteintech (Chicago, IL) and; Na+K+ ATPase was obtained from Santa Cruz (Dallas, TX). Anti-ROM1 antibody, anti-cone arrestin and anti-CNGB1 were gifts of Dr. Muna I. Naash (University of Oklahoma), Dr. Cheryl M. Craft (University of Southern California), and Dr. Martin Biel (Center for Drug Research, Institute of Pharmacology, Germany), respectively.

### PSC preparation

Mouse PSC was prepared essentially as described^10^,^64^. Briefly, fresh mouse eyes were dissected and retinas were placed in a tube with 150 μl of 8% OptiPrep (Nycomed, Oslo, Norway) prepared in Ringer’s buffer (130 mM NaCl, 3.6 mM KCl, 2.4 mM MgCl_2_, 1.2 mM CaCl_2_, 10 mM HEPES, pH 7.4, containing 0.02 mM EDTA) and vortexed for 1 min. The samples were centrifuged at 200 × g for 1 min, and the supernatant containing the PSC was transferred to fresh eppendorf tube. The resultant pellet was dissolved in 150 μl of 8% OptiPrep, vortexed, and centrifuged again at 200 × g for 1 min. These steps were repeated five times. The PSC was pooled (∼2 ml), overlaid on a 10–30% continuous gradient of OptiPrep in Ringer’s buffer, and centrifuged for 50 min at 26,500 × g. PSC were collected from second band (about two-thirds of the way from the top), diluted three times with Ringer’s buffer, and centrifuged for 3 min at 500 × g to remove the cell nuclei. The supernatant containing PSC was transferred to a new tube and centrifuged for 30 min at 26,500 × g. The pelleted material contained pure intact PSC.

### Immunofluorescence and Immunoblotting

For staining of the isolated PSC complex preparations, 10 μl of the suspension was spotted on glass slides and fixed in 4% paraformaldehyde in PBS (pH 7.4) for 15 minutes. Mouse retina was processed for immunofluorescence as described^26^,^65^,^66^. For staining with anti-PSMD3 and anti-IDE antibodies, consistent results were obtained when retinas were processed using paraffin-embedding^65^,^66^. Slides containing PSC and mouse retinal sections were washed with PBS, permeabilized, and blocked for 1 hour with blocking solution containing 5% normal goat serum with 0.5% Triton X-100 in PBS in a humidifying chamber at RT. Primary antibodies were added and slides were incubated overnight at 4 °C. After washing three times with PBS, samples were incubated for 1 hour with goat anti-rabbit (or mouse) Alexa Fluor 488 nm or 546 nm secondary antibody at room temperature. Hoechst 33342 (Life Technologies Corp.) was diluted with PBS to final 1 μg/mL and used to label the nuclei. Samples were washed with deionized water and then mounted (Fluoromount; Electron Microscopy Sciences, Hatfield, PA) under glass coverslips and visualized using a microscope (Leica TCS SP5 II laser; Leica Microsystems).

For Immunoblotting, equal amount of protein samples (50 μg) were denatured at 95^º^C for 5 min in 10 mm Tris-HCl, pH 6.8, 4% SDS, 20% sucrose, 4% β-mercaptoethanol and separated on SDS-polyacrylamide gels. Proteins were transferred onto Immobilon-FL membranes (Millipore, Bedford, MA). Blots were blocked in 5% nonfat milk in Tris-buffered saline containing 0.1% Tween 20 (TBST) for 30 min and labeled with the primary antibody in 5% nonfat milk prepared in TBST for overnight. The blots were washed with TBST and labeled with a secondary anti-mouse or anti-rabbit antibody conjugated to HRP and processed for chemiluminescence reaction.

### Proteomic Analysis

Analyses of three biological replicates were performed using 6 retinas each from wild type and *Rpgr*^*ko*^ mice (total 18 retinas from each genotype). Samples were run ~1.5 cm into the resolving gel of 10% minigels (BioRad, Hercules, CA) and protein-containing regions were excised, destained, and cut into 1 × 1 mm pieces. Gel pieces were then placed in 1.5-ml eppendorf tubes with 1 ml of water for 30 min. The water was removed and 100μl of 250 mM ammonium bicarbonate was added. For reduction 20 μl of a 45 mM solution of 1, 4 dithiothreitol (DTT) was added and the samples were incubated at 50 C for 30 min. The samples were cooled to room temperature and alkylated by adding 20 μl of a 100 mM iodoacetamide solution for 30 min. Gel slices were washed twice with 1 ml water aliquots. The water was removed and 1 ml of 50:50 (50 mM Ammonium Bicarbonate: Acetonitrile) was placed in each tube and samples were incubated at room temperature for 1 hr. The solution was then removed and 200 μl of acetonitrile was added to each tube at which point the gels slices turned opaque white. The acetonitrile was removed and gel slices were further dried in a Speed Vac. Gel slices were rehydrated in 50 μl of 2 ng/μl trypsin (Sigma) in 0.01% ProteaseMAX Surfactant (Promega): 50 mM Ammonium Bicarbonate. Additional bicarbonate buffer was added to ensure complete submersion of the gel slices. Samples were incubated at 37^o^C for 21hrs. The supernatant of each sample was then removed and placed in a separate 1.5 ml eppendorf tube. Gel slices were further dehydrated with 100 μl of 80:20 (Acetonitrile: 1% formic acid). The extract was combined with the supernatants of each sample. The samples were then dried down in a Speed Vac. Samples were dissolved in 25 μl of 5% Acetonitrile in 0.1% trifluroacetic acid prior to injection on LC/MS/MS.

### LC/MS/MS on Q Exactive

A 3 μl aliquot was directly injected onto a custom packed 2 cm x 100 μm C_18_ Magic 5 μ particle trap column. Peptides were then eluted and sprayed from a custom packed emitter (75 μm x 25 cm C_18_ Magic 3 μm particle) with a linear gradient from 95% solvent A (0.1% formic acid in water) to 35% solvent B (0.1% formic acid in Acetonitrile) in 90 minutes at a flow rate of 300 nl per minute on a Waters Nano Acquity UPLC system. Data dependent acquisitions were performed on a Q Exactive mass spectrometer (Thermo Scientific) according to an experiment where full MS scans from 300–1750 m/z were acquired at a resolution of 70,000 followed by 12 MS/MS scans acquired under HCD fragmentation at a resolution of 35,000 with an isolation width of 1.2 Da.

### Data Analysis

Raw data files were processed with Proteome Discoverer (version 1.3) prior to searching with Mascot Server (version 2.4) against the Uniprot_Mouse database. Search parameters utilized were fully tryptic with 2 missed cleavages, parent mass tolerances of 10 ppm and fragment mass tolerances of 0.05 Da. A fixed modification of carbamidomethyl cysteine and variable modifications of acetyl (protein N-term), pyro glutamic for N-term glutamine, and oxidation of methionine were considered. Search results were loaded into the Scaffold Viewer (Proteome Software, Inc.) for comparisons of sample results between wild type and *Rpgr*^*ko*^ PSC. Prior to additional analyses, major contaminants such as keratins, ribosomal and mitochondrial proteins were removed from our PSC proteome to increase the specificity of identified proteins.

We used previously reported stringency criteria to enrich specifically altered proteins. All MS/MS samples were analyzed using Mascot search engine (Matrix Science, London, UK; version 2.4.0). Mascot was set up to search the SwissProt_062613 database (selected for mice) with a fragment ion mass tolerance of 0.050 Da and a parent ion tolerance of 10.0 PPM. Scaffold (version Scaffold_4.3.4, Proteome Software Inc., Portland, OR) was used to validate MS/MS based peptide and protein identifications. Peptide identifications were accepted if they could be established at greater than 95.0% probability by the Peptide Prophet algorithm^67^,^68^ with Scaffold delta-mass correction. Protein identifications were accepted if they could be established at greater than 95.0% probability and contained at least 3 identified peptides. We put false discovery rate (FDR) cutoff ~<1, which means that more than 99% of the proteins in our list were true proteins.

### Quantitative proteomic analysis

Spectral counting has been used for differentiating the abundance of protein amount in complex mixture of different biological samples. However protein quantification by spectral counting would be biased by length of proteins because larger proteins generate more spectral counts as compared to smaller proteins. For our quantitative proteomic analysis, we adopted a previously described method called exponentially modified protein abundance index (emPAI)^69^. emPAI is widely used method in quantitative comparative proteomics and offers approximate, label-free, relative quantitation of the proteins in a mixture based on protein coverage by the peptide matches in a database search result. PAI (protein abundance index) of each protein was calculated by dividing number of observed peptides to the number of observable peptides per protein.
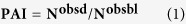
where N^obsd^ and N^obsbl^ are the number of unique observed peptides and the number of unique observable peptides per protein, respectively.

For absolute quantitation, PAI was converted to exponentially modified PAI (emPAI),
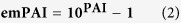
which is proportional to protein content in a protein mixture. Solubility prediction of identified proteins was calculated using Expropriator (http://mips.helmholtz-muenchen.de/proso/proso.seam)^70^.

### Functional Analysis

After finding proteins, which are differently expressed, we performed pathway analysis by using DAVID (Database for Annotation, Visualization and Integrated Discovery) classification system. We analyzed differential expression of proteins based on >2 fold change, for both down regulated and up regulated, from overall wild type and knockout datasets.

## Additional Information

**How to cite this article**: Rao, K. N. *et al.* Ablation of retinal ciliopathy protein RPGR results in altered photoreceptor ciliary composition. *Sci. Rep.* **5**, 11137; doi: 10.1038/srep11137 (2015).

## Supplementary Material

Supplementary Information

Supplementary Information

Supplementary Information

## Figures and Tables

**Table 1:** **Proteins with reduced abundance in PSC of** ***Rpgr*** ^***ko***^ **at 2 months.** S1, S2, and S3 are the three biological repeat samples used in the present study.

				**emPAI VALUES**	
**Identified Proteins**	**Accession Number**	**Molecular Weight**	**Fold Change**	**C57 Mean S1 S2 S3**	***Rpgr***^***ko***^**Mean S1 S2 S3**
N-myc downstream-regulated gene 1	NDRG1_MOUSE	43 kDa	0.2	0.343- 0.312, 0.411, 0.306	0.076-0.073, 0.059, 0.0966
Protein Tfg	Q9Z1A1_MOUSE	43 kDa	0.2	0.343- 0.276, 0.355, 0.398	0.076- 0.071, 0.053, 0.1048
SEC14like protein 2	S14L2_MOUSE	46 kDa	0.2	0.316- 0.358, 0.275, 0.315	0.071- 0.069, 0.084, 0.06
26S proteasome nonATPase regulatory subunit 3	PSMD3_MOUSE	61 kDa	0.2	0.234- 0.256, 0.145, 0.301	0.0539- 0.0566, 0.038, 0.066
EH domain containing protein 4	EHD4_MOUSE	61 kDa	0.2	0.23- 0.18, 0.36, 0.15	0.053-0.051, 0.0399, 0.0686
Ubiquitin carboxyl-terminal hydrolase	E9PYI8_MOUSE	52 kDa	0.3	0.44- 0.32, 0.56, 0.44	0.129-0.097, 0.250, 0.04
Golgi reassembly stacking protein 2, isoform CRA_c	A2ATI6_MOUSE	37 kDa	0.3	0.291- 0.213, 0.442, 0.218	0.0889-0.0811, 0.0690, 0.1166
Insulin degrading enzyme	F6RPJ9_MOUSE	114 kDa	0.3	0.288- 0.251, 0.290, 0.323	0.088- 0.079, 0.080, 0.105
Septin2	SEPT2_MOUSE	42 kDa	0.3	0.257- 0.222, 0.321, 0.228	0.0793- 0.072, 0.063, 0.1022
Bardet-Biedl syndrome 4 protein homolog	BBS4_MOUSE	58 kDa	0.4	0.315- 0.306, 0.467, 0.172	0.116- 0.107, 0.120, 0.121
Isoform 2 of Transmembrane emp24 domain-containing protein 10	TMEDA_MOUSE	11 kDa	0.4	1.88- 1.89, 1.54, 2.21	0.698- 0.666, 0.723, 0.705
Cullin3	CUL3_MOUSE	89 kDa	0.4	0.287- 0.267, 0.345, 0.249	0.114- 0.108, 0.114, 0.12
Farnesyl pyrophosphate synthase	FPPS_MOUSE	41 kDa	0.4	0.598- 0.549, 0.668, 0.577	0.264- 0.237, 0.212, 0.343
26S protease regulatory subunit 10B	PRS10_MOUSE	44 kDa	0.4	0.538- 0.556, 0.413, 0.645	0.24- 0.18, 0.27, 0.27
T-complex protein 1 subunit zeta	TCPZ_MOUSE	58 kDa	0.4	1.04- 1.02, 1.06, 1.04	0.469- 0.447, 0.525, 0.435
GTP-binding nuclear protein Ran	RAN_MOUSE	24 kDa	0.5	1.45- 1.33, 1.59, 1.43	0.667- 0.612, 0.734, 0.655
Proliferation-associated protein 2G4	PA2G4_MOUSE	44 kDa	0.5	0.337- 0.333, 0.411, 0.267	0.156- 0.149, 0.155, 0.164
ATP-binding cassette subfamily F member 1	ABCF1_MOUSE	95 kDa	0.5	0.31- 0.29, 0.3, 0.34	0.145- 0.116, 0.178, 0.141
Guanine nucleotide-binding protein G(T) subunit gammaT1	GBG1_MOUSE	9 kDa	0.5	14.5- 13.8, 14.2, 15.5	6.8- 5.3, 6.9, 8.2
Prom1 protein	Q8R056_MOUSE	94 kDa	0.5	1.04- 1.03, 1.01, 1.08	0.502- 0.489, 0.623, 0.394
Glutathione S transferase P 1	GSTP1_MOUSE	24 kDa	0.5	1.87- 1.77, 1.82, 2.02	0.933- 0.899, 0.929, 0.971
Coatomer subunit beta	COPB_MOUSE	107 kDa	0.5	0.391- 0.313, 0.405, 0.455	0.197- 0.177, 0.2, 0.214
Isoform 2 of ADP ribosylation factor-like protein 6	ARL6_MOUSE	22 kDa	0.5	2.61- 2.57, 2.16, 3.1	1.36- 1.22, 1.48, 1.38
Isoform 3 of Glyoxalase domain containing protein 4	GLOD4_MOUSE	31 kDa	0.5	0.667- 0.606, 0.689, 0.706	0.359- 0.348, 0.377, 0.352
Guanine nucleotide-binding protein G(I)/G(S)/G(T) subunit beta1	GBB1_MOUSE	37 kDa	0.5	30.9- 31.4, 30.2, 31.1	16.7- 15.9, 16.3, 17.9
Exportin2	XPO2_MOUSE	110 kDa	0.5	0.418- 0.402, 0.471, 0.381	0.226- 0.197, 0.228, 0.253
Cullin5	CUL5_MOUSE	91 kDa	0.5	0.28- 0.23, 0.3, 0.31	0.151- 0.146, 0.162, 0.145
26S proteasome non-ATPase regulatory subunit 1	PSMD1_MOUSE	106 kDa	0.5	0.237- 0.241, 0.209, 0.261	0.129- 0.116, 0.131, 0.14

**Table 2:** **Proteins with reduced abundance in PSC of** ***Rpgr*** ^***ko***^ **at 4 months.** S1, S2, and S3 are the three biological repeat samples used in the present study.

				**emPAI VALUES**	
**Identified Proteins**	**Accession Number**	**Molecular Weight**	**Fold Change**	**C57Mean S1 S2 S3**	***Rpgr***^***ko***^**Mean S1 S2 S3**
Fascin2	FSCN2_MOUSE	55 kDa	0	0.403- 0.409, 0.41, 0.39	0
Vesicle trafficking protein SEC22a	SC22A_MOUSE	35 kDa	0	0.307- 0.298, 0.311, 0.312	0
Insulin degrading enzyme	IDE_MOUSE	118 kDa	0.1	0.48- 0.46, 0.59, 0.39	0.0556- 0.0601, 0.0567, 0.05
Oral facial digital syndrome 1 protein homolog	OFD1_MOUSE	117 kDa	0.1	0.412- 0.409, 0.410, 0.417	0.0565- 0.0557, 0.0578, .056
Cullin5	CUL5_MOUSE	91 kDa	0.2	0.452- 0.448, 0.465, 0.443	0.109- 0.112, 0.098, 0.117
GTP-binding protein 1	GTPB1_MOUSE	72 kDa	0.3	0.531- 0.54, 0.529, 0.524	0.138- 0.141, 0.137, 0.136
Ubiquitin conjugating enzyme E2 O	UBE2O_MOUSE	141 kDa	0.3	0.227- 0.219, 0.236, 0.226	0.0701- 0.0698, 0.0709, 0.0696
Prolactin inducible protein	PIP_HUMAN	17 kDa	0.3	1.25- 1.248, 1.252, 1.25	0.409- 0.4089, 0.412, 0.4061
Jouberin	AHI1_MOUSE	120 kDa	0.3	0.335- 0.334, 0.336, 0.3349	0.111- 0.109, 0.112, 0.112
Potassium voltage gated channel subfamily V member 2	KCNV2_MOUSE	64 kDa	0.3	0.464- 0.467, 0.463, 0.462	0.156- 0.158, 0.157, 0.153
Protein transport protein Sec24C	SC24C_HUMAN	118 kDa	0.3	0.406- 0.398, 0.411, 0.409	0.141- 0.140, 0.143, 0.14
WD repeat-containing protein 35	WDR35_MOUSE	134 kDa	0.4	0.355- 0.35, 0.3539, 0.3551	0.124- 0.125, 0.122, 0.125
Vacuolar protein sorting associated protein 26B	VP26B_MOUSE	39 kDa	0.4	0.473- 0.471, 0.475, 0.473	0.169- 0.168, 0.170, 0.169
SH3containing GRB2-like protein 3-interacting protein 1	SGIP1_PSAOB	88 kDa	0.4	0.42- 0.419, 0.421, 0.42	0.152- 0.153, 0.1521, 0.1509
Proliferation-associated protein 2G4	PA2G4_MOUSE	44 kDa	0.4	0.611- 0.609, 0.612, 0.612	0.233- 0.234, 0.299, 0.166
ATP-binding cassette subfamily F member 1	ABCF1_MOUSE	95 kDa	0.4	0.433- 0.432, 0.435, 0.432	0.177- 0.179, 0.176, 0.176
Coiled-coil and C2 domain containing protein 2A	C2D2A_MOUSE	188 kDa	0.4	0.714- 0.709, 0.711, 0.722	0.297- 0.298, 0.297, 0.296
Nck-associated protein 1	NCKP1_MOUSE	129 kDa	0.4	0.31- 0.309, 0.311, 0.313	0.13- 0.11, 0.134, 0.146
F-actin capping protein subunit beta	CAPZB_MOUSE	31 kDa	0.4	0.739- 0.74, 0.7385, 0.7385	0.33- 0.329, 0.3401, 0.3209
AP3 complex subunit mu2	AP3M2_MOUSE	47 kDa	0.5	0.474- 0.472, 0.474, 0.4746	0.216- 0.215, 0.2161, 0.2169
WD40 repeat-containing protein SMU1	SMU1_MOUSE	58 kDa	0.5	0.25- 0.239, 0.259, 0.252	0.114- 0.113, 0.115, 0.114
26S proteasome non-ATPase regulatory subunit 3	PSMD3_MOUSE	61 kDa	0.5	0.233- 0.232, 0.234, 0.2321	0.108- 0.1079, 0.1085, 0.1076
Glutathione S transferase P 1	GSTP1_MOUSE	24 kDa	0.5	2.53- 2.529, 2.535, 2.526	1.18- 1.185, 1.178, 1.177
TBC1 domain family member	TBC24_MOUSE	63 kDa	0.5	0.407- 0.411, 0.406, 0.4031	0.213- 0.211, 0.214, 0.214
Voltage-dependent anion selective channel protein 1	VDAC1_RAT	31 kDa	0.5	9.26- 9.248, 9.259, 9.273	4.88- 4.85, 4.91, 4.88
WD repeat containing protein 7	WDR7_MOUSE	163 kDa	0.5	0.56-0 .568, 0.557, 0.555	0.297- 0.299, 0.3, 0.292
Cytoplasmic dynein 2 heavy chain 1	DYHC2_MOUSE	492 kDa	0.5	0.769- 0.768, 0.774, 0.765	0.413- .4123, .3998, .4269
Phosducin	PHOS_MOUSE	28 kDa	0.5	5.96- 5.971, 5.993, 5.916	3.2- 3.214, 3.189, 3.197
Kinesin like protein KIF1A	KIF1A_MOUSE	192 kDa	0.5	0.429- .4289, .431, .4271	0.232- 0.2324, 0.2295, 0.2241
UPF0568 protein C14orf166 homolog	CN166_MOUSE	28 kDa	0.5	0.68- 0.679, 0.686, 0.675	0.37- 0.365, 0.375, 0.37

**Table 3:** **Proteins with increased abundance in PSC of** ***Rpgr*** ^***ko***^ **at 2 months.** S1, S2, and S3 are the three biological repeat samples used in the present study.

				**emPAI VALUES**	
**Identified Proteins**	**Accession Number**	**Molecular Weight**	**Fold Change**	**C57Mean S1 S2 S3**	***Rpgr***^***ko***^**Mean S1 S2 S3**
Ubiquitin thio-esterase OTUB1	D3YWF6_MOUSE	28 kDa	INF	0	0.565- 0.521, 0.577, 0.597
26S proteasome non-ATPase regulatory subunit 7	PSMD7_MOUSE	37 kDa	INF	0	0.296- 0.278, 0.3, 0.31
Isoform 5 of Dynamin1like protein	DNM1L_MOUSE	24 kDa	3.4	7.77- 7.31, 7.68, 8.32	26.7- 27.1, 25.7, 27.3
Isoform 1 of Gammaadducin	ADDG_MOUSE	75 kDa	3	0.136- 0.145, 0.131, 0.132	0.404- 0.399, 0.413, 0.4
Potassium/sodium hyperpolarization activated cyclic nucleotide gated channel 1	HCN1_MOUSE	102 kDa	2.9	0.0986- 0.0924, 0.0999, 0.1035	0.285- 0.263, 0.278, 0.314
WD repeat containing protein 1	WDR1_MOUSE	66 kDa	2.6	0.212- 0.195, 0.225, 0.216	0.542- 0.431, 0.558, 0.637
Isoform 2 of Disks large homolog 1	DLG1_MOUSE	100 kDa	2.5	0.101- 0.099, 0.115, 0.089	0.253- 0.229, 0.261, 0.269
Isoform 2 of Putative tyrosine protein phosphatase auxilin	AUXI_MOUSE	99 kDa	2.5	0.103- 0.089, 0.110, 0.11	0.256- 0.247, 0.273,0.248
Thioredoxin related transmembrane protein 2	D3Z2J6_MOUSE	30 kDa	2.4	0.376- 0.352, 0.4, 0.376	0.892- 0.886, 0.879, 0.911
Ras-related protein Rab2A	RAB2A_MOUSE	24 kDa	2.4	0.939- 0.894, 1, 0.923	2.29- 2.16, 2.88, 1.83
Serine/threonine protein phosphatase 2A 56 kDa regulatory subunit epsilon isoform	2A5E_MOUSE	55 kDa	2.3	0.337- 0.265, 0.351, 0.395	0.789- 0.667, 0.8, 0.9
Isoform 2 of Chondroitin sulfate proteoglycan 5	CSPG5_MOUSE	57 kDa	2.2	0.182- 0.166, 0.190, 0.19	0.396- 0.382, 0.4, 0.406
Annexin A6	ANXA6_MOUSE	76 kDa	2.1	0.59- 0.46, 0.62, 0.69	1.23- 1.20, 1.37, 1.12
Isoform 3 of Neuronal cell adhesion molecule	NRCAM_MOUSE	139 kDa	2.1	0.0969- 0.0917, 0.099, 0.1	0.203- 0.210, 0.179, 0.22
Isoform 3 of Cell adhesion molecule 2	CADM2_MOUSE	44 kDa	2.1	0.665- 0.659, 0.7, 0.636	1.4- 1.09, 1.6, 1.51
Vesicle-associated membrane protein 2	B0QZN5_MOUSE	18 kDa	2	2.34- 2.19, 2.86, 1.97	4.6- 3.9, 4.8, 5.1
Septin7	E9Q1G8_MOUSE	51 kDa	2	0.552- 0.592, 0.488, 0.576	1.12- 1.08, 1.2, 1.08

**Table 4:** **Proteins with increased abundance in PSC of** ***Rpgr*** ^***ko***^ **at 4 months.** S1, S2, and S3 are the three biological repeat samples used in the present study.

				**emPAI VALUES**	
**Identified Proteins**	**Accession Number**	**Molecular Weight**	**Fold Change**	**C57Mean S1 S2 S3**	***Rpgr***^***ko***^**Mean S1 S2 S3**
Collectrin	TMM27_MOUSE	25 kDa	INF	0	1.32- 1.28, 1.31, 1.37
Ermin	ERMIN_MOUSE	32 kDa	INF	0	0.979- 0.989, 0.982, 0.966
Ras GTPase activating like protein IQGAP2	IQGA2_MOUSE	181 kDa	8.2	0.116- 0.115, 0.120, 0.113	0.945- 0.95, 0.943, 0.942
Ras GTPase activating like protein IQGAP1	IQGA1_MOUSE	189 kDa	5.7	0.0731- 0.0729, 0.073, 0.0732	0.415- 0.413, 0.445, 0.387
Sarcolemmal membrane associated protein	SLMAP_MOUSE	97 kDa	5	0.258- 0.2581, 0.259, 0.2569	1.29- 1.285, 1.295, 1.29
Na(+)/H(+) exchange regulatory cofactor NHERF1	NHRF1_MOUSE	39 kDa	3	0.383- 0.381, 0.3829, 0.3851	1.14- 1.13, 1.15, 1.14
Integrin alpha M	ITAM_MOUSE	127 kDa	3	0.223- 0.221, 0.224, 0.224	0.676- 0.675, 0.679, 0.674
Integrin alpha V	ITAV_MOUSE	115 kDa	3	0.491- 0.5, 0.490, 0.483	1.5- 1.48, 1.51, 1.51
Secretory carrier associated membrane protein 3	SCAM3_MOUSE	38 kDa	2.9	0.278- 0.279, 0.2789, 0.2761	0.793- 0.791, 0.794, 0.794
Beta-soluble NSF attachment protein	SNAB_MOUSE	34 kDa	2.8	0.974- .975, .9768, .9702	2.69- 2.7, 2.689, 2.681
Chondroitin sulfate proteoglycan 4	CSPG4_MOUSE	252 kDa	2.8	0.38- 0.37, 0.395, 0.375	1.05- 1.1, 1.04, 1.01
Radixin	RADI_BOVIN	69 kDa	2.7	1.41- 1.399, 1.415, 1.416	3.74- 3.76, 3.749, 3.711
Podocalyxin	PODXL_MOUSE	53 kDa	2.7	0.266- .265, .2657, .2673	0.716- 0.7149, 0.717, 0.7161,
Chondroitin sulfate proteoglycan 5	CSPG5_MOUSE	60 kDa	2.6	0.497-0.498, 0.495, 0.498	1.31- 1.3, 1.299, 1.331
Proteasome subunit alpha type1	PSA1_MOUSE	30 kDa	2.5	0.366- 0.365, 0.368, 0.365	0.909- 0.911, 0.908, 0.908
Sphingomyelin phosphodiesterase 2	NSMA_MOUSE	47 kDa	2.5	0.321- 0.322, 0.323, 0.319	0.799- 0.798, 0.7989, 0.8001
Gamma-soluble NSF attachment protein	SNAG_MOUSE	35 kDa	2.4	0.428- 0.427, 0.429, 0.428	1.04- 1.038, 1.041, 1.041
Tectonin beta-propeller repeat containing protein 1	TCPR1_MOUSE	130 kDa	2.2	0.162- 0.163, 0.161, 0.162	0.351- 0.3509, 0.3512, 0.3509
Alpha-soluble NSF attachment protein	SNAA_MOUSE	33 kDa	2.2	1.47- 1.465, 1.478, 1.467	3.2- 3.11, 3.25, 3.24
Myoferlin	MYOF_MOUSE	233 kDa	2.1	0.135- 0.136, 0.134, 0.1349,	0.282- 0.2781, 0.28201, 0.2878
PlexinB2	PLXB2_MOUSE	206 kDa	2.1	0.243- 0.2425, 0.244, 0.2425	0.511- 0.51, 0.513, 0.51
Vacuolar protein sorting-associated protein 35	VPS35_MOUSE	92 kDa	2	0.154- 0.155, 0.1529, 0.1541	0.303- 0.3, 0.299, 0.31
Ras-related protein Rab14	RAB14_HUMAN	24 kDa	2	1.22- 1.219, 1.24, 1.201	2.43- 2.41, 2.45, 2.43
CAAX prenyl protease 1 homolog	FACE1_MOUSE	55 kDa	2	0.259- 0.257, 0.261, 0.259	0.53- 0.519, 0.538, 0.533

**Table 5:** **Soluble proteins with altered in abundance in** ***Rpgr*** ^***ko***^ **PSC.** No predicted post-translational modifications to allow membrane association were detected in these proteins. MW: predicted molecular weight; kDa: kilo Daltons.

**2 months**				**4 months**			
**Decreased**		**Increased**		**Decreased**		**Increased**	
**Name**	**MW (kDa)**	**Name**	**MW (kDa)**	**Name**	**MW (kDa)**	**Name**	**MW (kDa)**
NDRG1	43	DLG1	100	IDE	118	IQGAP1	**189**
S14L2	46	AUX1	99	ABCF1	95	IQGAP2	**181**
PSMD3	61			CC2D2A	188	SLMAP	**97**
EHD4	61			GSTP1	24	SNAB	**34**
E9PYI8	52			PHOS	28	SNAG	**35**
A2ATI6	37			CN166	28	SNAA	**33**
F6RPJ9	114					TCPR1	**130**
BBS4	58			**AVERAGE**	**80**	**AVERAGE**	99.8
FPPS	41						
ABCF1	95						
GBG1	9						
GSTP1	24						
GLOD4	31						
**AVERAGE**	**51.7**	**AVERAGE**	**99.5**				
