# Supplementary material for: Ablation of retinal ciliopathy protein RPGR results in altered photoreceptor ciliary composition
Source: Sci Rep. 2015 Jun 11;5:11137. doi: 10.1038/srep11137 (PMC4463945; doi:10.1038/srep11137)

Ablation of retinal ciliopathy protein RPGR results in altered photoreceptor ciliary composition

Kollu N. Rao[1], Linjing Li[1], Manisha Anand[1], and Hemant Khanna[1*]

Supplementary Figure S1: Reduced abundance proteins: MS/MS data from *Rpgrko* and wild type PSC was analyzed to identify proteins with reduced abundance in *Rpgr*^{ko} PSC at 2 months (upper panel) and 4 months (lower panel) of age. Pathways represented by the identified proteins are represented here.

PROTEINS WITH REDUCED ABUNDANCE IN *Rpgr*^{ko} PSC AT 2 MONTHS OF AGE

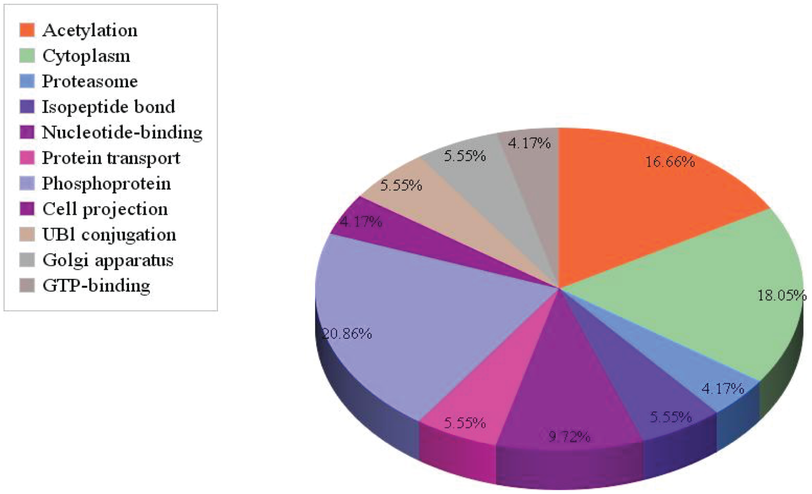

PROTEINS WITH REDUCED ABUNDANCE IN *Rpgr*^{ko} PSC AT 4 MONTHS OF AGE

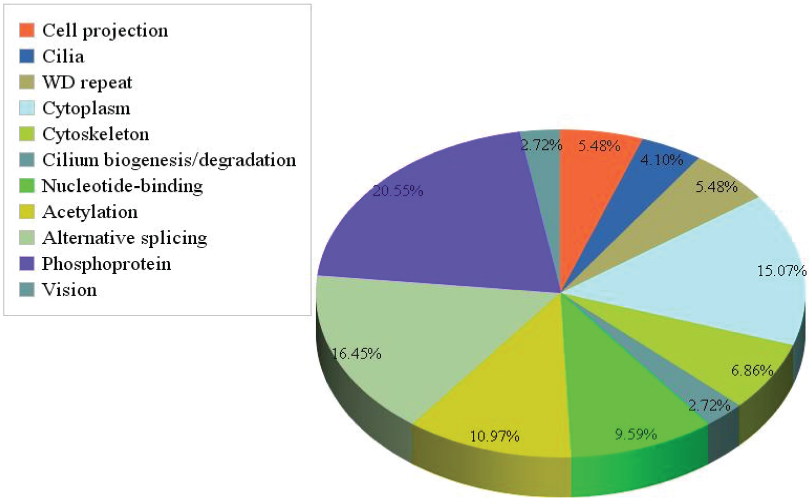

Supplementary Figure S2: Increased abundance proteins: MS/MS data from *Rpgrko* and wild type PSC was analyzed to identify proteins with increased abundance in *Rpgr*^{ko} PSC at 2 months (upper panel) and 4 months (lower panel) of age. Pathways represented by the identified proteins are represented here.

PROTEINS WITH INCREASED ABUNDANCE IN *Rpgr*^{ko} PSC AT 2 MONTHS OF AGE

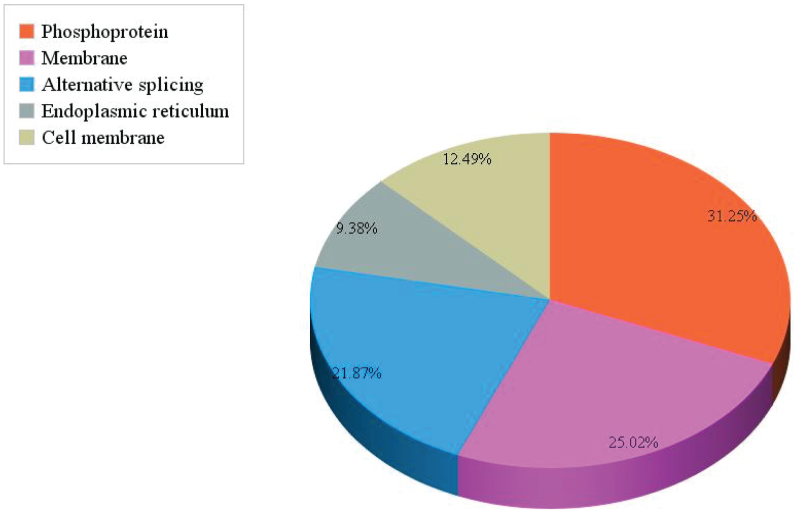

PROTEINS WITH INCREASED ABUNDANCE IN *Rpgr*^{ko} PSC AT 4 MONTHS OF AGE

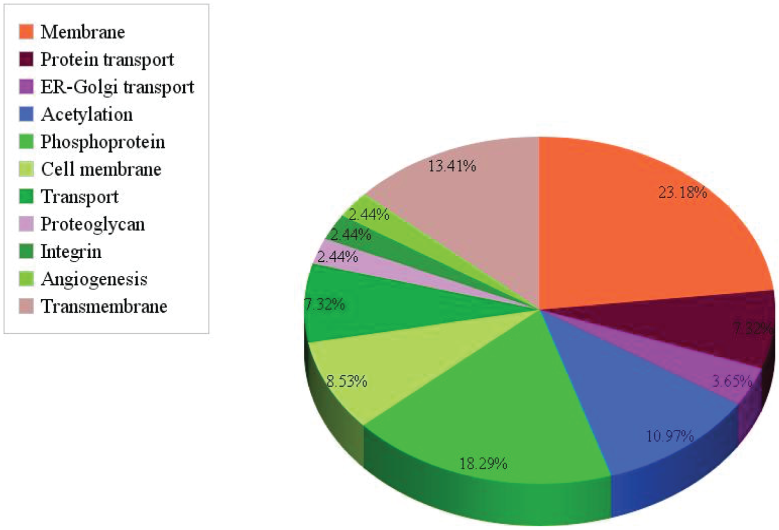

Supplementary Figure S3: Wild type retinal sections were stained with anti-acetylated a-tubulin (AcT; red), anti-γ-tubulin (basal body marker; red) or detyrosinated tubulin (DeTyr; green; TZ and microtubule marker) and IDE (**A**), OFD1 (**B**) or IQGAP1 (**C**). Merge (yellow) shows co-localization of the tested antibodies at the inner segment (IS) or TZ (transition zone). IDE staining was detected primarily in the IS and OPL (outer plexiform layer). At higher magnification (See Figure 4), IDE staining can be detected in the TZ. Arrows in inset (**B**) indicate co-localization of OFD1 and γ-tubulin. Arrows in C indicate a predominant localization of IQGAP1 at the tip of the TZ. Scale bar: 10 μm. OLM: outer limiting membrane; ONL: outer nuclear layer; OS: outer segment.

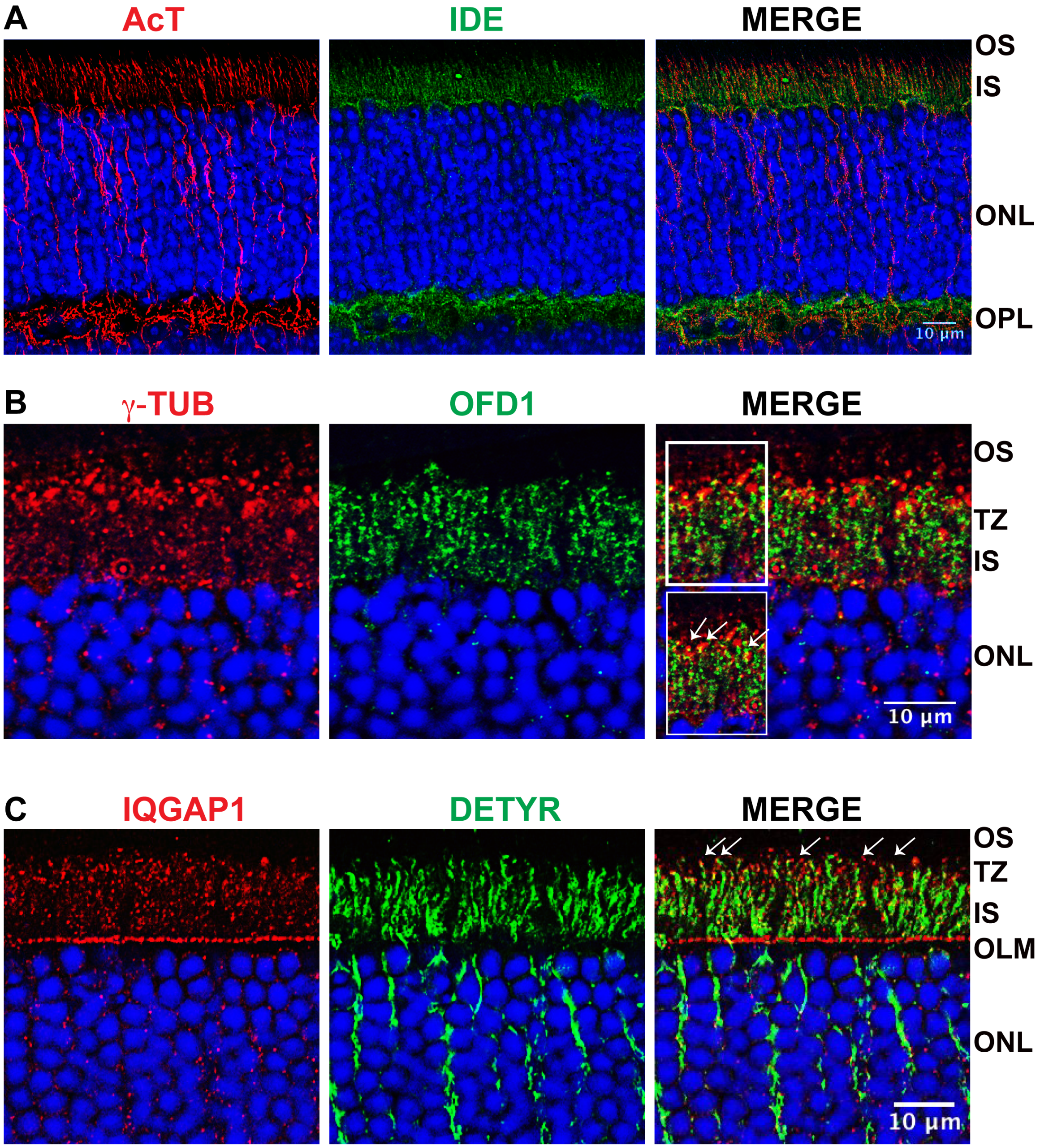

Supplement: Supplementary Information [file srep11137-s1.pdf]
